# Assessment of burnout in nursing technicians in intensive care exclusively during the COVID-19 pandemic

**DOI:** 10.1590/0034-7167-2024-0191

**Published:** 2025-11-28

**Authors:** Liana Amorim Corrêa Trotte, José Luís Guedes dos Santos, Etiane de Oliveira Freitas, Maria Gefé da Rosa Mesquita, Giulia Gazineo Trindade Assis, Leticia Luiza Cardoso Franco, Marluci Andrade Conceição Stipp

**Affiliations:** I Universidade Federal do Rio de Janeiro. Rio de Janeiro, Rio de Janeiro, Brazil; II Universidade Federal de Santa Catarina. Florianópolis, Santa Catarina, Brazil; III Universidade Federal de Santa Maria. Santa Maria, Rio Grande do Sul, Brazil

**Keywords:** Burnout, Professional, Nursing, COVID-19 Pandemic, Intensive Care Units, Mental Health., Burnout, Enfermería, COVID-19, Unidad de Cuidados Intensivos, Salud Mental.

## Abstract

**Objectives:**

to assess burnout syndrome in nursing technicians working in an Intensive Care Unit exclusively for COVID-19 patients.

**Methods:**

a quantitative and qualitative study was carried out at a university hospital in Rio de Janeiro, Brazil. The quantitative stage included a sample of 51 professionals, using Maslach Burnout Inventory. The qualitative stage involved interviews with 12 professionals, and data were analyzed using the Statistical Package of the Social Sciences and the R Interface for Multidimensional Analysis of Texts and Questionnaires.

**Results:**

most participants, mainly women (74.5%), did not present criteria for burnout. Quantitatively, protection against COVID-19, satisfaction and adequate resources reduced exhaustion and depersonalization. Qualitatively, factors for the development of burnout and protective aspects emerged.

**Conclusions:**

the association between emotional exhaustion, high work demands and lack of appropriate equipment suggests a greater predisposition to mental health problems. However, workers developed strategies that allowed them to measure their job satisfaction.

## RESUMEN

**Objetivos:**

evaluar el síndrome de burnout en técnicos de enfermería en una Unidad de Cuidados Intensivos exclusiva para pacientes con COVID-19.

**Métodos:**

estudio cuantitativo-cualitativo, realizado en un hospital universitario de Río de Janeiro, Brasil. La etapa cuantitativa incluyó una muestra de 51 profesionales, utilizando el *Maslach Burnout Inventory*. La etapa cualitativa consistió en entrevistas a 12 profesionales y los datos fueron analizados mediante el *Statistical Package of the Social Sciences* y el programa *R Interface pour les Analyses Multidimensionnelles de Textes et de Questionnaires*.

**Resultados:**

la mayoría de los participantes, principalmente mujeres (74,5%), no presentaron criterios de burnout. Cuantitativamente, la protección contra la COVID-19, la satisfacción y los recursos adecuados redujeron el agotamiento y la despersonalización. Cualitativamente emergieron factores para el desarrollo del burnout y aspectos protectores.

**Conclusiones:**

la asociación entre agotamiento emocional, alta demanda laboral y falta de equipamiento apropiado sugiere una mayor predisposición a problemas de salud mental. Sin embargo, los trabajadores desarrollaron estrategias que les permitieron medir su satisfacción laboral.

## INTRODUCTION

The coronavirus disease 2019 (COVID-19) pandemic has been characterized as one of the largest international public health diseases that has caused psychological and social disturbances that have affected the coping capacity of the entire society at different levels of intensity and spread^(1)^.

Several areas of knowledge, including psychology, have been called upon to develop emergency efforts and propose approaches to deal with the context of mental health. When addressing mental health, it is essential to highlight that it is a health domain characterized by its polysemy and plurality related to population groups’ mental state. These are highly complex conditions that transcend the mere absence of disease. This entire context has an impact on people’s feelings and can significantly alter their quality of life^(2)^.

Before the COVID-19 pandemic, mental health in the Americas was neglected, with systems and services facing a lack of public investment. The pandemic has exacerbated this situation, increasing demand for mental healthcare services while disrupting access to them. There is now an urgent need to expand and improve these services to ensure adequate health monitoring and prevention of future problems^(3)^.

Among the various psychological problems that healthcare professionals can develop, burnout syndrome (BS) stands out, which negatively affects physical and mental health, in addition to the presence of feelings and disorders such as fear, hopelessness, insomnia, post-traumatic stress disorder, exhaustion, anxiety and depression^(4)^.

The pandemic has led to the need for a rapid response from health agencies in an attempt to mitigate the damage caused and save as many lives as possible. Not only in our country, but around the world, it was necessary to create beds in exclusive Intensive Care Units (ICUs) due to the relentless outcome caused by the new virus. Considering the above, the greatest difficulty for the Human Resources department of hospitals involved in the fight against COVID-19 was finding professionals with the technical capacity to provide quality and safe care to patients who required intensive care during their treatment^(5)^.

In this context, nursing, a profession comprising the majority of healthcare professionals worldwide, was already facing significant challenges in working conditions before the pandemic. However, these challenges have been exacerbated by the COVID-19 pandemic, resulting in excessive workloads, lack of adequate rest, delayed salaries and lack of psychosocial support, factors that have also contributed to the high number of infected professionals^(6)^.

The Pan American Health Organization has proposed measures to mitigate these problems and their consequences, such as expanding professional training, ensuring adequate working hours and fair pay as well as overtime and sick leave when necessary^(7)^. However, the unpredictability of the evolution of the outbreak of a little-understood infectious disease, characterized by rapid spread and with the potential to have serious impacts on public health, contributed to a significant increase in physical and mental disorders in the general population, with a greater representation of BS, mainly in healthcare professionals^(8)^.

It is important to note that, in order to provide quality nursing care free from harm, professionals need to receive theoretical and practical support that enables them to perform their duties properly, especially in highly complex environments such as the ICU. However, this need was not met in more than 50% of healthcare professionals, as shown in a study carried out in Brazil during the pandemic^(9)^.

## OBJECTIVES

To assess BS in nursing technicians in an ICU exclusively for patients with COVID-19.

## METHODS

### Ethical aspects

To comply with ethical aspects, the study followed recommendations of Resolution 466/12, which approves guidelines and regulatory standards for research involving human beings, and Resolution 510/2016 of the Brazilian National Health Council, which presents recommendations for health research conducted online. The project was approved by the Research Ethics Committee.

### Study design

This is a study with a quantitative-qualitative approach. In this strategy, quantitative and qualitative data collection occurred simultaneously, followed by statistical analysis, in order to generalize the results with a larger sample, or to converge quantitative and qualitative data, to expand the research problem^(10)^.

### Study location

The study setting was the ICU of a public university hospital in the city of Rio de Janeiro, located in southeastern Brazil, created exclusively to care for patients with COVID-19 during the pandemic period.

### Population and sample

The unit studied had a total of 96 nursing professionals, distributed among 30 nurses and 66 nursing technicians, who worked on a 12x60-hour work shift. The quantitative study was of the analytical cross-sectional type, with a sample composed of 51 mid-level nursing professionals (nursing technicians). The qualitative stage was descriptive in nature and included the participation of 12 nursing technicians.

The decision to highlight nursing technicians in this study considered that this professional category is the most vulnerable, due to excess demand, low autonomy and limited social support, which is in line with Karesek’s theory^(11)^. According to the author, low social control in work environments contributes to the development of chronic diseases, such as cardiovascular and mental illnesses, and this occurs due to the physiological dysregulation caused by the overload and difficulty of self-regulation of the organism in the face of high demands and reduced control over the work performed^(11)^.

Nursing technicians who were performing their duties in direct care of patients with COVID-19 and had been employed in the unit for at least three months were included. Professionals who were away from care work and who performed their duties in the remote or administrative mode were excluded.

### Instruments and data collection - quantitative stage

At this stage of the research, a personal and professional characterization form and the Maslach Burnout Inventory (MBI) were used^(12)^.

The personal and professional characterization form addressed personal (age, sex and marital status) and professional characteristics (professional training, length of experience in the profession, length of direct work with COVID-19, work shift, length of work in the unit and institution, weekly working hours and existence of another employment relationship).

MBI was adapted and validated for Brazilian culture, and aims to measure professional burnout by assessing your feelings about your work^(12,13)^. This is a self-administered instrument with 22 statements. BS assessment is based on three independent but interrelated dimensions, such as emotional exhaustion, depersonalization and professional accomplishment. Emotional exhaustion corresponds to physical exhaustion and emotional exhaustion in dealing with stressful situations. Depersonalization is the tendency of workers to assess themselves negatively, becoming unhappy and dissatisfied with their professional development. Reduced personal accomplishment involves the development of cold, negative and insensitive attitudes towards the recipients of a service provided.

Items are scored according to the frequency with which professionals experience certain situations: (1) never; (2) a few times a year; (3) a few times a month; (4) a few times a week; and (5) daily^(14)^. To assess the results, the sum of participants’ responses for each subscale must be obtained.

To analyze the level of BS, the percentile cutoff was calculated to establish the low, moderate, and high levels. In the emotional exhaustion dimension, the level of burnout is considered low when it is equal to or less than 18, moderate when it is between 19 and 24, and high when it is equal to or greater than 25. In relation to depersonalization, a level of burnout is classified as low if it is less than or equal to 6, moderate if it is in the range of 7 to 9, and high if it is greater than or equal to 10. Regarding reduced personal accomplishment, a level of burnout is identified as low if it is equal to or greater than 32, moderate if it is between 28 and 31, and high if it is equal to or less than 28.

It is important to highlight that in the emotional exhaustion and depersonalization subscales, the higher the score, the greater the feeling of emotional exhaustion and depersonalization perceived by healthcare professionals. In the professional accomplishment subscale, higher scores indicate high professional accomplishment.

Data collection was carried out from April to June 2021. Potential participants were informed about the study by the nursing manager and invited to participate. They were then given links to access the online questionnaire through the Google Forms^®^ survey platform. Participants could complete the questionnaire via computer or smartphone. Upon accessing the website, participants were first directed to a page containing information about the purpose of the study, anonymity, and confidentiality. They were then given access to the Informed Consent Form. If they agreed to participate, after reading it, they clicked on the “affirmative” option and were directed to the data collection page.

### Quantitative result analysis

Data were analyzed using the Statistical Package of the Social Sciences version 25.0. Descriptive statistics were used to determine the distribution of sociodemographic characteristics, including sex, age, race, length of direct care for COVID-19 patients, length of professional experience in years, and so on. Categorical variables were represented by absolute and relative frequency. Quantitative variables were represented by mean and standard deviation and by median and interquartile range (median [25; P75]), according to the distribution verified by the Shapiro-Wilk normality test. To compare MBI scores, the chi-square or Mann-Whitney tests were used. A two-sided p-value less than 0.05 was considered statistically significant in the analyses.

### Instrument and data collection - qualitative stage

The qualitative stage was conducted through semi-structured in-person interviews, which addressed questions related to factors at work that contributed to BS. They were conducted in person, since one of the researchers was involved in the professional training of workers at this hospital. Participants were selected by lottery, observing the proportionality between work shifts (day and night). The interviews were conducted before the start of the work shift, or after it according to participants’ availability, who, in prior contact, chose the hospital itself as the ideal place to conduct the interviews. The interviews lasted on average 20 minutes each, and were conducted in a room reserved exclusively for data collection.

The number of interviewees was defined based on participants’ adherence to the research, following the following data saturation criteria^(15)^: base size, defining the initial number of three interviews analyzed to identify main topics; round length, which determined the need for three additional rounds with three interviews each to verify the emergence of new topics; and limit of new information, which established a maximum percentage of new topics accepted before considering saturation reached less than 5% for moderate saturation, and 0% for total saturation. In the present study, total saturation was reached after 12 interviews were conducted. Participants were identified by Arabic numerals corresponding in random sequence to the interviews (1, 2, and so on).

### Qualitative result analysis

The text *corpus* generated through the interviews was subjected to two analytical approaches, carried out using the free software *Interface de R pour les Analyses Multidimensionnelles de Textes et de Questionnaires* (IRaMuTeQ^®^), which were carefully chosen by the researcher, in line with the theoretical-methodological objectives of this research: lexicographic analysis and Descending Hierarchical Classification (DHC). These approaches were integrated to provide a more detailed understanding of results obtained and an assessment of the coding process carried out by IRaMuTeQ^®(16,17)^.

This procedure of assessing lexicons transforms linguistic content, allowing the conversion of data into binary or dichotomous variables, whose indicators are used to quantify and represent the characteristics. DHC encodes the text *corpus*, segmenting it into text segments (TSs), which are classified according to their vocabulary and frequency of word roots. A utilization rate of over 75% of TSs is considered satisfactory for analysis. DHC allows these segments to be grouped into classes with homogeneous vocabulary, using correlation logic. The identification of these classes makes it possible to infer predominant concepts and content in the text *corpus*, aiding in data interpretation^(16,17)^.

## RESULTS

In the quantitative stage, 51 nursing technicians participated in the study from a population of 66 professionals. In the sample studied, 74.5% were female; 64.3% declared themselves to be brown or black; 84.3% reported having high school/technological education as their highest qualification; 68.6% developed their activities during the day shift. The research participants had an average age of 39.9 (±9.69) years.

In BS assessment among participants, the prevalence of each dimension of MBI was verified, which are presented in Table 1. It was observed that the majority of workers presented low exhaustion, low depersonalization and high professional accomplishment. However, it is important to highlight that, when the average cut-off point of the instrument was assessed, there was a percentage greater than 30% of workers who presented moderate emotional exhaustion.

**Table 1:** Prevalence by dimensions of the Maslach Burnout Inventory among nursing technicians, Rio de Janeiro, Rio de Janeiro, Brazil, 2022

Subscale		Burnout		
	Mean (SD)	Low	Moderate	High
Emotional exhaustion	**19.03(6.99)**	**29(56.9%)**	16(31.4%)	6(11.8%)
Depersonalization	**7.70 (3.61)**	**27(52.9%)**	13(25.5%)	11(21.6%)
Professional accomplishment	33.03 (5.94)	10(19.6%)	4(7.8%)	**37(72.5%)**

Table 2 presents the association between MBI dimensions and work-related variables. As can be seen, feeling protected against COVID-19 at work was associated with low levels of emotional exhaustion and depersonalization and high professional accomplishment. A good perception of the adequacy and quality of material and technological resources was associated with a low level of depersonalization. Furthermore, being satisfied with one’s current job was associated with a low level of emotional exhaustion.

**Table 2:** Association between Maslach Burnout Inventory dimensions and work-related variables, Rio de Janeiro, Rio de Janeiro, Brazil, 2022

	Emotional exhaustion				Professional accomplishment				Depersonalization			
	**Low** **n (%)**	**Moderate** **n (%)**	**High** **n (%)**	**P**	**Low** **n (%)**	**Moderate** **n (%)**	**High** **n (%)**	**p**	**Low** **n (%)**	**Moderate** **n (%)**	**High** **n (%)**	**p**
**Received training from the institution on COVID-19**												
YES	28 (60.9)	13 (28.3)	5 (10.9)	0.170	8 (17.4)	3 (6.5)	35 (76.1)	0.137	25 (54.3)	11 (23.9)	10 (21.7)	0.821
NO	1(20)	3 (60)	1 (20)		2 (40)	1 (20)	2 (40)		2 (40)	2 (40)	1 (20)	
**Do you feel protected against COVID-19 at work?**												
YES	28 (63.6)	13 (29.5)	3 (6.8)	**<0.001**	6 (13.6)	4 (9.1)	34 (77.3)	**0.003**	27 (61.4)	8 (18.2)	9 (20.5)	**0.002**
NO	1 (14.3)	3 (42.9)	3 (42.9)		4 (57.1)	0 (0)	3 (42.9)		0 (0)	5 (71.4)	2 (28.6)	
**The number of nursing professionals is adequate for the care provided**												
YES	21 (61.8)	10 (29.4)	3 (17.6)	0.478	5 (14.7)	2 (5.9)	27 (79.4)	0.316	20 (58.8)	8 (23.5)	6 (17.6)	0.457
NO	8 (47.1)	6 (35.3)	3 (8.8)		5 (29.4)	2 (11.8)	10 (58.8)		7 (41.2)	5 (29.4)	5 (29.4)	
**Are the material and technological resources adequate in number and quality?**												
YES	16 (76.2)	4 (19.0)	1 (4.8)	0.082	4 (19.0)	1 (4.8)	16 (76.2)	0.089	14 (66.7)	5 (23.8)	2 (9.5)	**0.016**
NO	13 (43.3)	12 (40.0)	5 (16.7)		6 (20.0)	3 (10.0)	21 (70.0)		13 (43.3)	8 (26.7)	9 (30.0)	
**How do you feel about your current job?**												
Dissatisfied	1 (20)	2 (40)	2 (40)	**0.043**	2 (40)	0 (0)	3 (60)	0.533	2 (40)	1 (20.0)	2 (40)	0.691
Satisfied	28 (60.9)	14 (30.4)	4 (8.7)		8 (17.4)	4 (8.7)	34 (73.9)		25 (54.3)	12 (26.1)	9 (19.6)	

**Figure 1:**
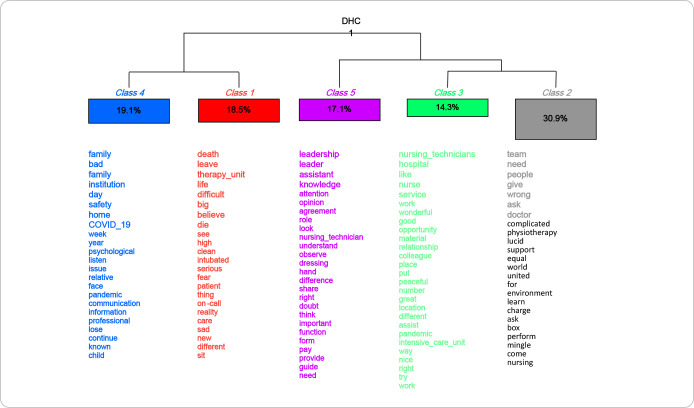
Dendrogram showing semantic classes and their relationships, Rio de Janeiro, Rio de Janeiro, Brazil, 2022

*Source: IRaMuTeQ^®^ Dendogramme DHC1 - phylogram.*

In the qualitative stage, 12 nursing technicians participated in the study. Through dendrogram analysis (Figure 1), it was possible to identify the lexical content of classes and the most frequent words in each class. Participants’ statements were initially organized into two categories. In category “1”, classes 1 and 4 were found, which were related to each other, forming the category entitled “Potential factors for the development of burnout”. In category “2”, classes 2, 3 and 5 were identified, which gave rise to the second category, “Protective aspects against burnout during the pandemic”.

From participants’ reports, it was possible to highlight experiences of workers during the pandemic that could contribute to emotional exhaustion, depersonalization, and sometimes even dissatisfaction. Factors such as patients’ clinical condition, unexpected and significant deaths in total numbers, and complex work dynamics proved to be different challenges to be overcome. These aspects contributed to the formation of the first category “Potential factors for the development of burnout”, as shown in Chart 1.

**Chart 1:** Potential factors for the development of burnout, Rio de Janeiro, Rio de Janeiro, Brazil, 2022

CLASSES	STATEMENTS	INFERENCES
Class 1 - Coping with the severity and loss of COVID-19 patients	*I arrived in the midst of chaos, where many people were dying. It was a very sad situation, a unique and desperate moment, and we didn’t know how to deal with it all.* (5) *Last year, I cried a lot. In my first week on the COVID-19 unit, we had eight deaths in the ICU, which only had 20 beds. It was horrible for me to have to face the reality of choosing who would live and who would die. It was very painful.* (2) *I never thought I would see people dying without even being able to say goodbye to their family members. The patient dies without seeing anyone, and that is one of the most horrible experiences I have ever had.* (6) *I wanted to cry, I wanted to run away, but necessity made me face the difficulties. I felt like giving up, but I decided to try. I thought, “I will try a little more, one day at a time”.* (10)	Having witnessed many deaths and dealing with the suffering of patients and their families faced with something unknown created psychological demands.
Class 4 - Work dynamics imposed by COVID-19	*Financial issues are a concern. We currently work under temporary contracts, and this directly impacts our personal lives and brings a lot of uncertainty.* (7) *The feeling was that we were facing a war. We knew it was a real battle, and the feeling of being in a war was constant.* (2) *Because it was a new disease that we were unfamiliar with, it was very difficult to adapt and learn the necessary precautions. It is hard, stressful work, and I consider it extremely difficult.* (1) *We cannot leave the sector during the shift. We stay from 7 a.m. until 12 p.m. without drinking water or going to the bathroom. After lunch, we stay until 7 p.m., without being able to leave again.* (10) *We end up dehydrated and with our health compromised. I get home, after 24 hours of work, completely parched from thirst, with pain in my kidneys and an irregular bowel movement due to not having ingested liquids. I need to recover quickly to face the next day, which is difficult from a physiological point of view.* (9) *Now, doctors have to choose which patients they want to use for certain sedation drugs. This makes us very upset. It is distressing to see a patient who has a good chance of recovery but cannot receive the appropriate treatment because resources are limited and they have to choose another one.* (8)	Exhausting workdays and the need to provide precise and specific care for the treatment of COVID-19 were factors that required great effort from professionals. In addition, the work dynamics, which required adaptation to physiological needs due to the high possibility of viral transmission, contributed to emotional exhaustion and depersonalization.

The second category, “Protective aspects against burnout during the pandemic”, described in Chart 2, is composed of situations experienced by participants, which suggest professional accomplishment. Workers revealed satisfaction with working conditions and relationships within the team, as well as recognition of the importance of their work and the need for it to be performed with quality, considering clinical aspects and their biopsychosocial-spiritual needs.

**Chart 2:** Protective aspects of burnout during the pandemic, Rio de Janeiro, Rio de Janeiro, Brazil, 2022

CLASS	STATEMENTS	INFERENCES
Class 3 - Availability of resources to ensure better patient care and worker protection	*What I didn’t mention was our safety. Very few nursing technicians contracted COVID-19 during this one year of work, almost no one was infected.* (3) *At first, we were very afraid of the disease. We didn’t know much about it and we didn’t know if we would have adequate protective equipment. Everyone arrived scared. Later, we realized that there was enough clothing and all the necessary medications. This was a great relief, because when we deal with people, we need to have the appropriate means to work. Thank God, we had these resources.* (6) *I believe that the availability of everything, from bed linen to supplies, makes all the difference. It may seem like a simple thing, but it directly influences the quality and safety of care. For instance, what if a patient needs to be aspirated and there is no catheter available?* (1)	Workers expressed satisfaction with the availability of resources to provide quality care to patients and with institutional strategies to protect workers. This generated feelings of appreciation and professional accomplishment.
Class 5 - The sensitivity of the professional to provide care to the patient with COVID-19	*Most patients are very anxious, and this is something we often observe. This anxiety often causes saturation to drop. However, in some cases, a simple conversation, conveying security and calm, is enough to reassure the patient and improve the situation.* (12) *It is essential to show affection for the patient, especially when they are waking up. At this time, they need even more care and attention, as they are away from their family, without seeing familiar faces or having the presence of their family around.* (6) *Even when intubated and sedated, patients can still feel and hear words of comfort. We often say that everything will be fine, that it is just a phase and that they will soon be extubated. It is possible to notice that, upon hearing this, the patient shows slight emotion, as if they feel welcomed.* (4) *We take every care to prevent the patient from developing pressure ulcers, such as by repositioning them correctly. Body hygiene is carried out very carefully, and the patient’s skin is never left without moisture. This care is essential for well-being and recovery.* (1)	The recognition that the patient is a human being and requires quality care that meets all of his/her needs, contributing to less depersonalization of the worker and greater personal accomplishment.
Class 2 - The quality of intra and interprofessional relationships	*The nursing technicians, nurses, and cleaning staff are an excellent team. I really like the work environment. In addition, the supply of materials and the structure offered by the hospital are very good, which makes our work easier.* (3) *The relationship with the team is great. We always try to do everything together, in a collaborative way.* (4) *The team members are very competent, always looking to learn and contribute. When we need help, we just have to ask, because everyone is helpful and committed. It is a very close-knit and efficient team that works very well.* (5) *My colleagues are very patient and teach me the right way to work. I have no complaints about the team. Everyone is wonderful. Since it is a new disease, we learn something different every day, which makes the job a constant learning experience.* (12)	Teamwork and the quality of relationships between team members were satisfactory. This perception by participants helped to mitigate the negative impacts of the pandemic, minimizing emotional exhaustion and igniting feelings of accomplishment.

## DISCUSSION

This study demonstrated that most nursing technicians did not meet the criteria for developing BS, according to assessment carried out by MBI. In quantitative data, only the variable feeling protected against COVID-19 stood out as a protective factor against the development of BS. In qualitative data, the interviews made it possible to identify other factors that protected them and facilitated the development of BS. The application of two research methods allowed a more detailed analysis of the phenomenon, facilitating the interpretation of results obtained. It was observed that protective factors stood out in relation to risk factors, which may explain the low levels of emotional exhaustion and depersonalization and high level of professional accomplishment in the sample studied.

In results collected at the beginning of the pandemic, it was already possible to identify that the most frequent feeling among health workers was fear. Negative perceptions related to mental health were linked to insomnia, psychological distress, burnout, anxiety and depressive symptoms^(18)^.

A study conducted with 12,596 Chinese nurses, also conducted at the beginning of the pandemic, showed that the average scores for each of the three MBI subscales were between low and moderate, supporting the results of this study in relation to the emotional exhaustion and depersonalization domains. However, with regard to the professional accomplishment subscale, the Chinese reported low scores, which was not observed in our study^(19)^.

It is worth noting that, in general, studies show that work in intensive care settings contributes to the overload of the emotional and physical domains, due to the complex work interventions carried out by the nursing team, which can cause imbalances between their needs, values and activities they perform. In other words, the high workload and the lack of time for patient care are factors that contribute to the occurrence of emotional disorders and low job satisfaction^(20)^. However, in this research, high levels of job accomplishment were found, highlighting that the feeling of protection against the virus, the availability of resources to provide quality care to patients, combined with institutional strategies to protect workers, generated feelings of appreciation.

To cope with the adverse activities inherent to the pandemic context, the institution’s nursing management recommended that professionals seek individual tools that would prevent further damage to their health, highlighting, among them, the development of resilience. Studies show that developing resilience at work acts as a protective effect in the presence of high levels of emotional exhaustion and depersonalization, domains with the highest burnout scores^(21,22)^.

A study carried out with nurses in the Philippines highlighted that good teamwork development was an important factor in increasing professionals’ resilience and the drive to overcome challenges in the face of the pandemic^(23)^. In this study, this factor was also highlighted as protective, demonstrated by the feeling of good quality of inter and intraprofessional relationships, which helped to minimize unfavorable factors for BS.

After a prolonged period of the COVID-19 pandemic, two years after its onset, a significant incidence of nurses at risk of developing burnout was observed as well as a greater propensity to consider leaving their jobs. The work environment quality was the main factor related to the intention to leave work and to the rates of emotional exhaustion of Belgian and Turkish nurses working in ICUs during the pandemic. Furthermore, research also indicated a positive correlation between the presence of anxiety or depression and high levels of burnout^(24,25)^. However, this study demonstrated that the main contributing factors to the development of BS in the Brazilian institution studied were related to the implications of direct patient care.

Many studies have pointed out factors that directly influence high rates of burnout, such as lack of personal protective equipment, fear of being infected, increased mortality rates compared to normal, successive waves of increased contamination, societal expectations and accumulation of activities over time^(26-29)^. In this study, the following stood out as potential factors that could induce burnout: emotional issues related to living with the serious context in which the patients found themselves, physical issues associated with exhausting working hours and the need for adaptation at work, which limited the accomplishment of basic human needs.

The relationship between the professional/patient ratio contributes to professional accomplishment, and has already been demonstrated in Brazilian studies. When investigating the professional accomplishment of nurses and nursing technicians working in ICUs, nursing technicians had better scores in professional accomplishment, compared to nurses, and the latter were responsible for seven patients, while nursing technicians were responsible for two patients^(30,31)^. These findings may have been supported in this study, since there was a similar professional/patient ratio in the studied setting. However, this relationship was not verified.

A recent scoping review identified protective and triggering factors for the development of BS in nurses working in ICUs during the COVID-19 pandemic. Increased workload, lack of equipment, social stigma, and fear of contagion were the main risk factors. Protective factors highlighted were aspects such as social support from leaders and colleagues, professional recognition, use of personal protective equipment, and witnessing the successful recovery of patients^(32)^, given that these factors are analogous to the context studied.

### Study limitations

The limitations of the study were related to convenience sampling, as it was developed in a single hospital in a large center and belonging to the university. In addition to this, the fact that it was a cross-sectional observational study that measured variables at only one specific moment in time limited its generalizability is also a limitation.

### Contributions to health

Due to the relevance for the quality of health work, managers, institutions and professionals must pay attention to the practice of strategies to confront BS that need to be present from senior management, with the development of institutional policies of support and administrative responsibility, middle management with tactical plans for supervision and guidance of managers as well as strategic plans of leadership for identification and mentoring of professionals.

## CONCLUSIONS

The findings of this study demonstrated that the association between emotional exhaustion, high work demands and lack of appropriate equipment suggests a greater predisposition to problems that affect mental health. Nevertheless, workers were able to develop strategies that allowed them to measure their satisfaction with the work performed. It is suggested that additional studies be developed to explore the relationships between potential factors and protective factors for the development of BS.

## Data Availability

The research data are available only upon request.
