# Supplementary material for: Assessment of burnout in nursing technicians in intensive care exclusively during the COVID-19 pandemic
Source: Rev Bras Enferm. 2025 Nov 28;78(5):e20240191. doi: 10.1590/0034-7167-2024-0191 (PMC12673610; doi:10.1590/0034-7167-2024-0191)
Supplement: Translation

# Translation: Avaliação do *burnout* em técnicos de enfermagem em terapia intensiva exclusiva na pandemia de COVID-19

**DOI:** 10.1590/0034-7167-2024-0191pt

**Authors:** Liana Amorim Corrêa Trotte, José Luís Guedes dos Santos, Etiane de Oliveira Freitas, Maria Gefé da Rosa Mesquita, Giulia Gazineo Trindade Assis, Leticia Luiza Cardoso Franco, Marluci Andrade Conceição Stipp

**Affiliations:** I Universidade Federal do Rio de Janeiro. Rio de Janeiro, Rio de Janeiro, Brasil; II Universidade Federal de Santa Catarina. Florianópolis, Santa Catarina, Brasil; III Universidade Federal de Santa Maria. Santa Maria, Rio Grande do Sul, Brasil

**Keywords:** Burnout, Enfermagem, COVID-19, Unidade de Terapia Intensiva, Saúde Mental.

## Abstract

**Objetivos:**

avaliar a síndrome de *burnout* em técnicos de enfermagem de Unidade de Terapia Intensiva exclusiva para pacientes com COVID-19.

**Métodos:**

estudo quanti-qualitativo, realizado em hospital universitário no Rio de Janeiro, Brasil. A etapa quantitativa incluiu uma amostra de 51 profissionais, utilizando-se o *Maslach Burnout Inventory*. A etapa qualitativa envolveu entrevistas com 12 profissionais, e os dados foram analisados pelo *Statistical Package of the Social Sciences* e *Interface de R pour les Analyses Multidimensionnelles de Textes et de Questionnaires*.

**Resultados:**

a maioria dos participantes, principalmente mulheres (74,5%), não apresentou critérios para *burnout*. Quantitativamente, proteção contra COVID-19, satisfação e recursos adequados reduziram exaustão e despersonalização. Qualitativamente, emergiram fatores para desenvolvimento do *burnout* e aspectos protetores.

**Conclusões:**

a associação entre exaustão emocional, alta demanda de trabalho e falta de equipamentos apropriados sugere maior predisposição a problemas de saúde mental. No entanto, os trabalhadores desenvolveram estratégias que permitiram medir sua satisfação com trabalho.

## INTRODUÇÃO

A pandemia da doença do coronavírus 2019 (COVID-19) caracterizou-se como uma das maiores doenças de saúde pública internacional que causou distúrbios psicológicos e sociais que afetaram a capacidade de enfrentamento de toda a sociedade em diversos níveis de intensidade e propagação^(1)^.

Diversas áreas do conhecimento, incluindo a psicologia, foram chamadas para desenvolver esforços emergenciais e propor abordagens para lidar com o contexto da saúde mental. Ao abordar a questão da saúde mental, é fundamental destacar que se trata de um domínio da saúde caracterizado pela sua polissemia e pluralidade relacionado ao estado mental de grupos populacionais. São condições de alta complexidade que transcendem a mera ausência de doenças. Todo esse contexto repercute nos sentimentos das pessoas, e pode alterar significativamente a sua qualidade de vida^(2)^.

Antes da pandemia de COVID-19, a saúde mental na Região das Américas era negligenciada, com sistemas e serviços enfrentando escassez de investimento público. A pandemia agravou essa situação, aumentando a demanda por serviços de saúde mental enquanto interrompia o acesso a eles. Agora, há uma urgente necessidade de expansão e melhoria desses serviços para garantir um monitoramento adequado da saúde e prevenção de futuros problemas^(3)^.

Entre os vários problemas psíquicos que profissionais de saúde podem desenvolver, destaca-se a síndrome de burnout (SB), que afeta negativamente a saúde física e mental, além da presença de sentimentos e transtornos como medo, desesperança, insônia, transtorno de estresse pós-traumático, esgotamento, ansiedade e depressão^(4)^.

A pandemia ocasionou a necessidade de rápida resposta dos órgãos de saúde, na tentativa de amenizar os danos causados e de salvar o maior número de vidas possíveis. Não somente em nosso país, mas ao redor do mundo, foi necessária a criação de leitos em Unidades de Terapia Intensiva (UTIs) exclusivas, devido ao desfecho implacável causado pelo novo vírus. Em face ao exposto, a maior dificuldade do setor de Recursos Humanos dos hospitais envolvidos no combate ao COVID-19 foi encontrar profissionais com capacidade técnica de atender com qualidade e segurança os pacientes que necessitavam de cuidados intensivos durante seu tratamento^(5)^.

Neste contexto, a enfermagem, profissão composta pela maioria dos profissionais de saúde em todo o mundo, já enfrentava desafios significativos nas condições de trabalho antes da pandemia. No entanto, esses desafios se agravaram com a pandemia de COVID-19, resultando em cargas de trabalho excessivas, falta de descanso adequado, atrasos nos salários e falta de apoio psicossocial, fatores esses que também contribuíram para o alto número de profissionais contaminados^(6)^.

A Organização Pan-Americana de Saúde propôs medidas para mitigar esses problemas e suas consequências, como a ampliação da capacitação dos profissionais, garantia de uma jornada de trabalho adequada e pagamento justo, além de horas extras e licença médica quando necessário^(7)^. Porém, a imprevisibilidade da evolução do surto de uma doença infecciosa pouco compreendida, caracterizada por uma rápida propagação e com o potencial de ter graves impactos na saúde pública, contribuiu para aumento expressivo de distúrbios físicos e mentais na população em geral, com maior representatividade da SB, principalmente em profissionais de saúde^(8)^.

Salienta-se que, para uma assistência de enfermagem de qualidade e livre de danos, é necessário que profissionais recebam suporte teórico e prático que os habilitem para devida atuação, principalmente em ambientes altamente complexos como a UTI. Porém, essa necessidade não foi atendida em mais de 50% dos profissionais de saúde, conforme aponta o estudo realizado no Brasil durante a pandemia^(9)^.

## OBJETIVOS

Avaliar a SB em técnicos de enfermagem de uma UTI exclusiva para pacientes com COVID-19.

## MÉTODOS

### Aspectos éticos

Para atender aos aspectos éticos, foram seguidas as recomendações da Resolução nº 466/12, que aprova diretrizes e normas reguladoras de pesquisa envolvendo os seres humanos, e da Resolução nº 510/2016 do Conselho Nacional de Saúde, que apresenta as recomendações para as pesquisas em saúde realizadas *online.* O projeto foi aprovado pelo Comitê de Ética em Pesquisa de referência.

### Desenho do estudo

Trata-se de estudo com abordagem quanti-qualitativa. Nessa estratégia, a coleta de dados quantitativos e qualitativos ocorreu concomitantemente, seguindo de análise estatística, a fim de generalizar os resultados com uma amostra maior, ou convergir os dados quantitativos e qualitativos, para a ampliação do problema da pesquisa^(10)^.

### Local do estudo

O cenário do estudo foi a UTI de um hospital universitário público da cidade do Rio de Janeiro, situado na região Sudeste do Brasil, criada exclusivamente para o atendimento de pacientes com COVID-19 durante o período pandêmico.

### População e amostra

A unidade pesquisada possuía um total de 96 profissionais de enfermagem, distribuídos em 30 enfermeiros e 66 técnicos de enfermagem, que trabalhavam em escala de trabalho 12x60 horas. O estudo quantitativo foi do tipo transversal analítico, com uma amostra composta por 51 profissionais da enfermagem de nível médio (técnicos de enfermagem). A etapa qualitativa foi de caráter descritiva e contou com a participação de 12 técnicos de enfermagem.

A decisão de destacar os técnicos de enfermagem neste estudo considerou que essa categoria profissional é a mais vulnerável, devido ao excesso de demanda, à baixa autonomia e ao limitado apoio social, o que vai ao encontro da teoria de Karesek^(11)^. Segundo o autor, o baixo controle social em ambientes de trabalho contribui para o desenvolvimento de doenças crônicas, como cardiovasculares e mentais, e isso ocorre devido à desregulação fisiológica causada pela sobrecarga e dificuldade de autorregulação do organismo diante das demandas elevadas e controle reduzido sobre o trabalho exercido^(11)^.

Foram incluídos técnicos de enfermagem que estivessem exercendo suas funções no atendimento direto ao paciente com COVID-19 e estavam empregados na unidade há pelo menos três meses. Foram excluídos os profissionais que estavam em afastamento do trabalho assistencial e que exerciam suas funções na modalidade de trabalho remoto ou administrativo.

### Instrumentos e coleta de dados - etapa quantitativa

Nesta etapa da pesquisa, utilizaram-se uma ficha de caracterização pessoal e profissional e o instrumento *Maslach Burnout Inventory* (MBI)^(12)^.

A ficha de caracterização pessoal e profissional abordava características pessoais (idade, sexo e estado civil) e profissionais (formação profissional, tempo de experiência na profissão, tempo de trabalho direto com o COVID-19, turno de trabalho, tempo de trabalho na unidade e na instituição, carga horária semanal e existência de outro vínculo empregatício).

O MBI foi adaptado e validado para a cultura brasileira, e tem como objetivo mensurar o desgaste profissional por meio da avaliação do seu sentimento em relação ao seu trabalho^(12,13)^. Trata-se de instrumento autoaplicável, com 22 afirmações. A avaliação da SB ocorre a partir de três dimensões independentes, mas relacionadas entre si, como exaustão emocional, despersonalização e realização profissional. A exaustão emocional corresponde ao desgaste físico e esgotamento emocional para lidar com situações estressoras. A despersonalização é a tendência do trabalhador se autoavaliar de forma negativa, tornando-se infeliz e insatisfeito com o seu desenvolvimento profissional. A diminuição da realização pessoal envolve o desenvolvimento de atitudes frias, negativas e insensíveis direcionadas aos receptores de um serviço prestado.

Os itens são pontuados de acordo com a frequência que o profissional vivencia determinadas situações: (1) nunca; (2) algumas vezes no ano; (3) algumas vezes ao mês; (4) algumas vezes na semana; e (5) diariamente^(14)^. Para avaliação dos resultados, deve-se obter a soma das respostas dos participantes para cada subescala.

Para análise do nível da SB, realizou-se o cálculo de corte dos percentis para estabelecer os níveis baixo, moderado e alto. Na dimensão da exaustão emocional, o nível de *burnout* é considerado baixo quando é igual ou inferior a 18, moderado, quando está entre 19 e 24, e alto, quando é igual ou superior a 25. Em relação à despersonalização, um nível de *burnout* é classificado como baixo se for menor ou igual a 6, moderado, se estiver na faixa de 7 a 9, e alto, se for maior ou igual a 10. No que concerne à diminuição da realização pessoal, um nível de *burnout* é identificado como baixo se for igual ou superior a 32, moderado, se estiver entre 28 e 31, e alto, se for igual ou inferior a 28.

É importante ressaltar que nas subescalas exaustão emocional e despersonalização, quanto maior a pontuação, maior o sentimento de exaustão emocional e despersonalização percebida pelo profissional de saúde. Na subescala realização profissional, maiores pontuações referem uma alta realização profissional.

A coleta de dados foi realizada de abril a junho de 2021. Os potenciais participantes foram informados sobre o estudo pela chefia de enfermagem e convidados a participar e, em seguida, recebiam *links* de acesso ao questionário *online* através da plataforma de pesquisa *Google Forms^®^*. Os participantes poderiam preencher o questionário via computador ou smartphone. Ao acessar o site, os participantes eram direcionados primeiramente para uma página contendo informações sobre o objetivo do estudo, anonimato e confidencialidade. Em seguida, tinham acesso ao Termo de Consentimento Livre e Esclarecido. Caso concordassem em participar, após a leitura, clicavam na opção “afirmativa” e eram direcionados à página de coleta de dados.

### Análise dos resultados quantitativos

Os dados foram analisados no software *Statistical Package of the Social Sciences*, versão 25.0. Estatísticas descritivas foram usadas para determinar a distribuição das características sociodemográficas, incluindo sexo, idade, raça, tempo de atuação no cuidado direto a pacientes com COVID-19, tempo de experiência profissional em anos e assim por diante. As variáveis categóricas foram representadas por frequência absoluta e relativa. As variáveis quantitativas foram representadas por média e desvio padrão e pela mediana e intervalo interquartílico (mediana [25; P75]), de acordo com a distribuição verificada pelo teste de normalidade de Shapiro-Wilk. Para a comparação entre os escores do MBI, foram utilizados os testes qui-quadrado ou Mann-Whitney. Um valor de p bilateral inferior a 0,05 foi considerado estatisticamente significativo nas análises.

### Instrumento e coleta de dados - etapa qualitativa

A etapa qualitativa foi realizada por meio de entrevistas presenciais semiestruturadas, que abordavam perguntas relacionadas aos fatores no trabalho que contribuíam para SB. Foram realizadas em formato presencial, uma vez que uma das pesquisadoras estava envolvida com o treinamento profissional dos trabalhadores deste hospital. A seleção dos participantes foi realizada por sorteio, observando-se a proporcionalidade entre turnos de trabalho (diurno e noturno). As entrevistas foram realizadas antes do início do turno de trabalho, ou após o mesmo de acordo com a disponibilidade dos participantes, que, em contato prévio, escolheram o próprio hospital como local ideal para realização das mesmas. As entrevistas tiveram em média duração de 20 minutos cada, e foram realizadas em sala reservada exclusivamente para coleta de dados.

O número de entrevistados foi definido a partir da adesão dos participantes à pesquisa, obedecendo-se os seguintes critérios de saturação dos dados^(15)^: tamanho base, definindo o número inicial de três entrevistas analisadas para identificar temas principais; comprimento da rodada, que determinou a necessidade três rodadas adicionais com a realização de três entrevistas cada para verificar o surgimento de novos temas; e limite de nova informação, que estabeleceu um percentual máximo de novos temas aceitos antes de considerar a saturação atingida menor que 5%, para saturação moderada, e 0%, para saturação total. No presente estudo, a saturação total foi alcançada após a realização de 12 entrevistas. Os participantes foram identificados por número arábico correspondente em sequência aleatória à realização das entrevistas (1, 2 e assim sucessivamente).

### Análise dos resultados qualitativos

O *corpus* textual gerado através das entrevistas foi submetido a duas abordagens analíticas, realizadas por meio do *software* gratuito *Interface de R pour les Analyses Multidimensionnelles de Textes et de Questionnaires* (IRaMuTeQ^®^), que foram criteriosamente escolhidas pelo pesquisador, em consonância com os objetivos teórico-metodológicos da pesquisa: a análise lexicográfica e a Classificação Hierárquica Descendente (CHD). Essas abordagens foram integradas para proporcionar uma compreensão mais detalhada dos resultados obtidos e avaliação do processo de codificação realizado pelo IRaMuTeQ^®(16,17)^.

Esse procedimento de avaliar os léxicos transforma o conteúdo linguístico, permitindo a conversão dos dados em variáveis binárias ou dicotômicas, cujos indicadores são utilizados para quantificar e representar as características. Já a CHD codifica o *corpus* textual, segmentando-o em segmentos de texto (STs), que são classificados conforme seus vocabulários e frequência de raízes das palavras. Um índice de aproveitamento superior a 75% dos STs é considerado satisfatório para análise. A CHD permite agrupar esses segmentos em classes com vocabulários homogêneos, utilizando a lógica de correlação. A identificação dessas classes possibilita inferir concepções e conteúdos predominantes no *corpus* textual, auxiliando na interpretação dos dados^(16,17)^.

## RESULTADOS

Na etapa quantitativa, 51 técnicos de enfermagem participaram do estudo de uma população de 66 profissionais. Na amostra estudada, 74,5% eram do sexo feminino; 64,3% se declararam pardos ou pretos; 84,3% referiram ter como maior titulação o ensino médio/tecnológico; 68,6% desenvolviam suas atividades no turno diurno. Os participantes da pesquisa apresentaram uma idade média de 39,9 (±9,69) anos.

Na avaliação da SB entre os participantes, foi verificada a prevalência de cada dimensão do MBI, as quais estão apresentadas na Tabela 1. Observou-se que a maioria dos trabalhadores apresentou baixa exaustão, baixa despersonalização e alta realização profissional. Porém, não podemos deixar de destacar que, quando avaliada a média de corte do instrumento, houve um percentual maior que 30% de trabalhadores que apresentaram exaustão emocional de forma moderada.

**Tabela 1:** Prevalência por dimensões do *Maslach Burnout Inventory* entre técnicos de enfermagem, Rio de Janeiro, Rio de Janeiro, Brasil, 2022

Subescala		*Burnout*		
	Média (DP)	Baixo	Moderado	Alto
Exaustão emocional	**19,03(6,99)**	**29(56,9%)**	16(31,4%)	6(11,8%)
Despersonalização	**7,70 (3,61)**	**27(52,9%)**	13(25,5%)	11(21,6%)
Realização profissional	33,03 (5,94)	10(19,6%)	4(7,8%)	**37(72,5%)**

Na Tabela 2, foi apresentada a associação entre as dimensões do MBI e as variáveis relacionadas ao trabalho. Conforme pode-se observar, sentir-se protegido contra COVID-19 em seu trabalho estava associado à baixo nível de exaustão emocional e despersonalização e alta realização profissional. A boa percepção sobre a adequação e qualidade dos recursos materiais e tecnológicos foi associada ao baixo nível de despersonalização. Ainda, estar satisfeito com seu trabalho atual esteve associado ao baixo nível de exaustão emocional.

**Tabela 2:** Associação entre as dimensões do *Maslach Burnout Inventory* e variáveis relacionadas ao trabalho, Rio de Janeiro, Rio de Janeiro, Brasil, 2022

	Exaustão emocional				Realização profissional				Despersonalização			
	**Baixa** **n (%)**	**Moderada** **n (%)**	**Alta** **n (%)**	**P**	**Baixa** **n (%)**	**Moderada** **n (%)**	**Alta** **n (%)**	**p**	**Baixa** **n (%)**	**Moderada** **n (%)**	**Alta** **n (%)**	**p**
**Recebeu capacitação da instituição sobre COVID-19**												
SIM	28 (60,9)	13 (28,3)	5 (10,9)	0,170	8 (17,4)	3 (6,5)	35 (76,1)	0,137	25 (54,3)	11 (23,9)	10 (21,7)	0,821
NÃO	1(20)	3 (60)	1 (20)		2 (40)	1 (20)	2 (40)		2 (40)	2 (40)	1 (20)	
**Sente-se protegido contra COVID-19 em seu trabalho**												
SIM	28 (63,6)	13 (29,5)	3 (6,8)	**<0,001**	6 (13,6)	4 (9,1)	34 (77,3)	**0,003**	27 (61,4)	8 (18,2)	9 (20,5)	**0,002**
NÃO	1 (14,3)	3 (42,9)	3 (42,9)		4 (57,1)	0 (0)	3 (42,9)		0 (0)	5 (71,4)	2 (28,6)	
**O número de profissionais de enfermagem é adequado para a assistência prestada**												
SIM	21 (61,8)	10 (29,4)	3 (17,6)	0,478	5 (14,7)	2 (5,9)	27 (79,4)	0,316	20 (58,8)	8 (23,5)	6 (17,6)	0,457
NÃO	8 (47,1)	6 (35,3)	3 (8,8)		5 (29,4)	2 (11,8)	10 (58,8)		7 (41,2)	5 (29,4)	5 (29,4)	
**Os recursos materiais e tecnológicos estão adequados em número e qualidade?**												
SIM	16 (76,2)	4 (19,0)	1 (4,8)	0,082	4 (19,0)	1 (4,8)	16 (76,2)	0,089	14 (66,7)	5 (23,8)	2 (9,5)	**0,016**
NÃO	13 (43,3)	12 (40,0)	5 (16,7)		6 (20,0)	3 (10,0)	21 (70,0)		13 (43,3)	8 (26,7)	9 (30,0)	
**Como você se sente em relação ao seu trabalho atual?**												
Insatisfeito	1 (20)	2 (40)	2 (40)	**0,043**	2 (40)	0 (0)	3 (60)	0,533	2 (40)	1 (20,0)	2 (40)	0,691
Satisfeito	28 (60,9)	14 (30,4)	4 (8,7)		8 (17,4)	4 (8,7)	34 (73,9)		25 (54,3)	12 (26,1)	9 (19,6)	

Na etapa qualitativa, participaram do estudo 12 técnicos de enfermagem. Pela análise do dendrograma (Figura 1), foi possível identificar o conteúdo lexical das classes e os vocábulos mais frequentes em cada classe. Os depoimentos dos participantes foram inicialmente organizados em duas categorias. Na categoria “1”, encontraram-se as classes 1 e 4, que se relacionavam entre si, formando a categoria intitulada “Potenciais fatores para o desenvolvimento do *burnout*”. Já na categoria “2”, identificaram-se as classes 2, 3 e 5, que deram origem à segunda categoria, “Aspectos protetores ao *burnout* durante a pandemia”.

**Figura 1:** Dendrograma apresentando as classes semânticas e suas relações, Rio de Janeiro, Rio de Janeiro, Brasil, 2022

*Fonte: IRaMuTeQ^®^ Dendogramme CHD1 - phylogram.*

Pelo relato dos participantes, foi possível evidenciar vivências dos trabalhadores durante a pandemia que poderiam contribuir para exaustão emocional, despersonalização, trazendo, por vezes, até insatisfação. Fatores como a condição clínica dos pacientes, inesperadas e significativas mortes em números totais e uma dinâmica de trabalho complexa, mostraram-se como diferentes desafios a serem superados. Esses aspectos contribuíram para a formação da primeira categoria “Potenciais fatores para o desenvolvimento do *burnout*”, conforme apresentado no Quadro 1.

**Quadro 1:** Potenciais fatores para o desenvolvimento do burnout, Rio de Janeiro, Rio de Janeiro, Brasil, 2022

CLASSES	DEPOIMENTOS	INFERÊNCIAS
Classe 1 - O lidar com a gravidade e a perda dos pacientes com COVID-19	*Cheguei em meio a um caos, onde muitas pessoas morriam. Era uma situação muito triste, um momento único e desesperador, e nós não sabíamos como lidar com tudo aquilo.* (5) *No ano passado, chorei muito. Na minha primeira semana na unidade de COVID-19, tivemos oito mortes na UTI, que tinha apenas 20 leitos. Foi horrível para mim ter que enfrentar a realidade de escolher quem ia viver e quem ia morrer. Isso foi muito doloroso.* (2) *Eu nunca pensei que veria pessoas morrendo sem nem poder se despedir de seus familiares. O paciente morre sem ver ninguém, e isso é uma das experiências mais horríveis que já vivi.* (6) *Eu queria chorar, queria sair correndo, mas a necessidade me fez enfrentar as dificuldades. Tive vontade de desistir, mas decidi tentar. Pensei: “Vou tentar mais um pouco, um dia de cada vez”.* (10)	Ter presenciado muitas mortes e lidado com o sofrimento dos pacientes e familiares diante de algo desconhecido gerou demanda psicológica.
Classe 4 - Dinâmica do trabalho imposta pela COVID-19	*Questões financeiras são preocupantes. Atualmente, trabalhamos sob contratos temporários, e isso impacta diretamente nossa vida pessoal e traz muita incerteza.* (7) *A sensação era de que estávamos enfrentando uma guerra. Sabíamos que era uma batalha real, e o sentimento de estar em uma guerra era constante.* (2) *Por ser uma doença nova que não conhecíamos, foi muito difícil nos adaptarmos e aprendermos os cuidados necessários. É um trabalho árduo, tenso, e eu o considero extremamente pesado.* (1) *Não podemos sair do setor durante o turno. Ficamos das 7 horas da manhã até às 12 horas sem beber água ou ir ao banheiro. Depois do almoço, permanecemos até às 19 horas, sem poder sair novamente.* (10) *Acabamos desidratados e com a saúde prejudicada. Eu chego em casa, após 24 horas de trabalho, completamente seca de sede, com dor nos rins e o intestino desregulado por não ter ingerido líquidos. Preciso me recuperar rapidamente para enfrentar o próximo dia, o que é difícil do ponto de vista fisiológico.* (9) *Agora, os médicos têm que escolher quais pacientes vão usar determinados medicamentos de sedação. Isso nos deixa muito chateados. É angustiante ver um paciente com grandes chances de recuperação, mas que não pode receber o tratamento adequado porque os recursos são limitados e precisam ser escolhidos para outro.* (8)	Jornadas de trabalho exaustivas e a necessidade de execução de cuidados precisos e específicos para o tratamento da COVID-19 foram fatores que exigiram grande esforço dos profissionais. Além disso, as dinâmicas de trabalho, que demandaram adaptação quanto às necessidades fisiológicas devido à alta possibilidade de transmissão viral, contribuíram para a exaustão emocional e despersonalização.

A segunda categoria, “Aspectos protetores ao burnout durante a pandemia”, descrita no Quadro 2, é composta por situações vivenciadas pelos participantes, que sugerem realização profissional. Os trabalhadores revelaram satisfação quanto às condições de trabalho e relacionamento entre a equipe, bem como reconhecimento da importância do seu trabalho e necessidade do mesmo ser exercido com qualidade, considerando os aspectos clínicos e suas necessidades biopsicosocioespirituais.

**Quadro 2:** Aspectos protetores ao *burnout* durante a pandemia, Rio de Janeiro, Rio de Janeiro, Brasil, 2022

CLASSES	DEPOIMENTOS	INFERÊNCIAS
Classe 3 - Disponibilidade de recursos para garantir uma melhor assistência ao paciente e proteção do trabalhador	*O que eu não mencionei foi a nossa segurança. Muito poucos técnicos de enfermagem contraíram COVID-19 durante esse um ano de trabalho, quase ninguém foi infectado.* (3) *No início, tínhamos muito medo da doença. Não a conhecíamos direito e não sabíamos se teríamos equipamentos de proteção adequados. Todos chegaram assustados. Depois percebemos que havia paramentação suficiente e todas as medicações necessárias. Isso foi um grande alívio, porque, quando lidamos com pessoas, precisamos ter os meios adequados para trabalhar. Graças a Deus, nós tivemos esses recursos.* (6) *Acredito que a disponibilidade de tudo, desde a roupa de cama até os insumos, faz toda a diferença. Pode parecer algo simples, mas isso influencia diretamente a qualidade e a segurança do atendimento. Por exemplo, e se for necessário aspirar um paciente e não houver uma sonda disponível?* (1)	Os trabalhadores demonstraram sua satisfação com a disponibilidade de recursos para prestar assistência de qualidade aos pacientes e com as estratégias institucionais de proteção dos trabalhadores. Isso gerou sentimentos de valorização e realização profissional.
Classe 5 - A sensibilidade do profissional para prestar assistência ao paciente com COVID-19	*A maioria dos pacientes fica muito ansiosa, e isso é algo que observamos com frequência. Essa ansiedade, muitas vezes, faz com que a saturação caia. No entanto, em alguns casos, uma simples conversa, transmitindo segurança e calma, é suficiente para tranquilizar o paciente e melhorar a situação.* (12) *É essencial termos carinho pelo paciente, especialmente quando ele está despertando. Nessa hora, ele precisa de ainda mais cuidado e atenção, pois está longe da família, sem ver rostos conhecidos ou ter a presença de seus familiares por perto.* (6) *Mesmo intubados e sedados, os pacientes ainda conseguem sentir e ouvir palavras de conforto. Nós costumamos dizer que tudo vai ficar bem, que é apenas uma fase e que em breve serão extubados. É possível perceber que, ao ouvir isso, o paciente demonstra uma leve emoção, como se se sentisse acolhido.* (4) *Temos todo o cuidado para evitar que o paciente desenvolva úlceras de pressão, como ao reposicioná-lo corretamente. A higiene corporal é realizada de forma muito cuidadosa, e a pele do paciente nunca é deixada sem hidratação. Esses cuidados são essenciais para o bem-estar e a recuperação.* (1)	O reconhecimento de que o paciente é um ser humano e necessita de uma assistência de qualidade que atenda a todas as suas necessidades, contribuindo para menor despersonalização do trabalhador e maior realização pessoal.
Classe 2 - A qualidade das relações intra e interprofissionais	*Tanto os técnicos de enfermagem quanto os enfermeiros e o pessoal da higienização formam uma equipe excelente. Gosto muito do ambiente de trabalho. Além disso, o provimento de materiais e a estrutura oferecida pelo hospital são muito bons, o que facilita nosso trabalho.* (3) *A relação com a equipe é ótima. Procuramos sempre realizar tudo em conjunto, de forma colaborativa.* (4) *As pessoas da equipe são muito competentes, sempre buscando aprender e contribuir. Quando precisamos de ajuda, é só pedir, pois todos são solícitos e comprometidos. É uma equipe muito unida e eficiente, que funciona muito bem.* (5) *Os colegas têm muita paciência, ensinam o jeito certo de trabalhar, e não tenho nada a reclamar da equipe. Todos são maravilhosos. Como é uma doença nova, cada dia aprendemos algo diferente, o que torna o trabalho um constante aprendizado.* (12)	O trabalho em equipe e a qualidade das relações entre os membros que a compõem foram satisfatórios. Essa percepção por parte dos participantes foi um amenizador dos impactos negativos da pandemia, minimizando a exaustão emocional e acendendo sentimentos de realização.

## DISCUSSÃO

Esta pesquisa demonstrou que a maioria dos técnicos de enfermagem não apresentava critérios para o desenvolvimento da SB, de acordo com a avaliação realizada pelo MBI. Nos dados quantitativos, apenas a variável sentir-se protegido contra a COVID-19 se destacou como fator protetor contra o desenvolvimento da SB. Já nos dados qualitativos, as entrevistas possibilitaram identificar outros fatores que os protegiam e que facilitavam o desenvolvimento da SB. A aplicação de dois métodos de pesquisa permitiu uma análise mais detalhada do fenômeno, facilitando a interpretação dos resultados obtidos. Observou-se que os fatores protetores se destacaram em relação aos fatores de risco, o que pode explicar os baixos níveis de exaustão emocional e despersonalização e alto nível de realização profissional na amostra estudada.

Em resultados coletados no início da pandemia, já era possível identificar que o sentimento com maior frequência entre os trabalhadores da área da saúde era o medo. As percepções negativas relacionadas à saúde mental estavam atreladas à insônia, ao sofrimento psicológico, ao *burnout*, à ansiedade e aos sintomas depressivos^(18)^.

Estudo desenvolvido com 12.596 enfermeiros chineses, também realizado no início da pandemia, demonstrou que as notas médias para cada uma das três subescalas do MBI estavam entre baixo e moderado, corroborando os resultados deste estudo em relação aos domínios exaustão emocional e despersonalização. Porém, no que diz respeito à subescala de realização profissional, os chineses reportaram baixos índices, o que não foi observado em nosso estudo^(19)^.

Destaca-se que, em geral, estudos demonstram que o trabalho em cenários de cuidados intensivos contribui para a sobrecarga dos domínios emocional e físico, devido às complexas intervenções laborais realizadas pela equipe de enfermagem, podendo ocasionar desequilíbrios entre suas necessidades, valores e atividades que realizam. Ou seja, a alta carga de trabalho e a falta de tempo para o atendimento ao paciente são fatores que contribuem para ocorrência de transtornos emocionais e para baixa satisfação no trabalho^(20)^. No entanto, nesta pesquisa, foram encontrados índices de alta realização no trabalho, destacando que a sensação de proteção contra o vírus, a disponibilidade de recursos na prestação de assistência com qualidade aos pacientes, somados às estratégias institucionais de proteção aos trabalhadores, geraram sentimentos de valorização.

Para o enfrentamento das atividades adversas inerentes ao contexto pandêmico, foi indicado pela gestão de enfermagem da instituição que os profissionais buscassem ferramentas individuais que impedissem maiores danos à saúde, destacando-se, entre elas, o desenvolvimento da resiliência. Estudos evidenciam que desenvolver a capacidade de resiliência no trabalho atua como efeito protetor na presença de níveis elevados de desgaste emocional e despersonalização, domínios com maior pontuação de *burnout* ^(21,22)^.

Estudo realizado com enfermeiras nas Filipinas destacou que o bom desenvolvimento de trabalho em equipe foi um importante fator para aumentar a resiliência dos profissionais e o impulso para superar os desafios diante da pandemia^(23)^. Neste estudo, esse fator também foi destacado como protetor, demonstrado pela sensação de uma boa qualidade das relações inter e intraprofissionais, que auxiliavam na minimização de fatores desfavoráveis para SB.

Após tempo prolongado de pandemia de COVID-19, dois anos após o início, foi observada uma significativa incidência de enfermeiros em situação de risco para o desenvolvimento de *burnout* e, ainda, uma maior propensão a considerar a possibilidade de deixar seus empregos. A qualidade do ambiente de trabalho era o principal fator relacionado à intenção de deixar o trabalho e aos índices de esgotamento emocional de enfermeiros belgas e turcos que trabalhavam em UTI durante a pandemia. Além disso, as pesquisas também apontaram correlação positiva entre a presença de ansiedade ou depressão e altos níveis de burnout^(24,25)^. Todavia, neste estudo, demonstrou-se que os principais fatores contribuintes para desenvolvimento da SB na instituição brasileira pesquisada estavam relacionados às implicações do cuidado direto ao paciente.

Muitos estudos apontaram como fatores que influenciavam diretamente os altos índices de *burnout* aspectos como falta de equipamentos de proteção individual, medo de ser contaminado, aumento das taxas de mortalidade em relação ao normal, ondas sucessivas de maior contaminação, expectativas da sociedade e acúmulo de atividades ao longo do tempo^(26-29)^. Neste estudo, destacaram-se como potenciais fatores indutores de *burnout* as questões emocionais relacionadas ao convívio com o grave contexto nos quais os doentes se encontravam, questões físicas associadas às jornadas de trabalho exaustivas e à necessidade de adaptação no trabalho, que limitavam o atendimento das necessidades humanas básicas.

A relação entre a proporção profissional/paciente contribui para realização profissional, e já foi demonstrada em estudos brasileiros. Ao investigar a realização profissional de enfermeiros e técnicos de enfermagem atuantes em UTI, os técnicos de enfermagem possuíam melhores escores na realização profissional, comparados aos enfermeiros, sendo que esses eram responsáveis por sete pacientes, enquanto técnicos de enfermagem eram responsáveis por dois pacientes^(30,31)^. Tais achados podem ter sido corroborados neste estudo, uma vez que havia uma proporção profissional/paciente semelhante no cenário estudado. Todavia, essa relação não foi verificada.

Recente revisão de escopo levantou fatores protetores e disparadores para desenvolvimento da SB em enfermeiros que atuavam em UTIs durante a pandemia de COVID-19. O aumento da carga de trabalho, falta de equipamentos, estigma social e medo de contágio foram os principais fatores de risco. Como fatores protetores, foram destacados aspectos como apoio social de líderes e colegas, reconhecimento profissional, uso de ferramentas de proteção individual e testemunho da recuperação bem-sucedida dos pacientes^(32)^, dados esses análogos ao contexto pesquisado.

### Limitações do estudo

As limitações do estudo relacionaram-se à amostragem de conveniência, tendo sido desenvolvido em único hospital de um grande centro e pertencente à universidade. Além disso, o fato de ser um estudo observacional transversal que mede variáveis em apenas um momento específico ao longo do tempo limitou sua generalização também foi uma limitação.

### Contribuições para a área da saúde

Devido à relevância para a qualidade do trabalho em saúde, gestores, instituições e profissionais devem atentar-se para a prática de estratégias de enfrentamento da SB que precisam estar presentes desde a alta gestão, com o desenvolvimento de políticas institucionais de apoio e responsabilidade administrativa, média gestão com planos táticos de supervisão e orientação de gerentes, bem como planos estratégicos da liderança para identificação e mentoria dos profissionais.

## CONCLUSÕES

Os achados deste estudo demonstraram que a associação entre exaustão emocional, alta demanda de trabalho com falta de equipamentos apropriados sugere uma maior predisposição para problemas que afetem a saúde mental. Ainda assim, os trabalhadores foram capazes de desenvolver estratégias que permitiram mensurar sua satisfação pelo trabalho desenvolvido. Sugere-se que sejam desenvolvidos estudos adicionais que permitam explorar as relações entre os fatores potenciais e os fatores protetores para o desenvolvimento da SB.

## Data Availability

Os dados de pesquisa só estão disponíveis mediante solicitação.
